# Sperm Alert: Semen Quality Decline Linked to Ozone Pollution

**DOI:** 10.1289/ehp.114-a177

**Published:** 2006-03

**Authors:** Tanya Tillett

Some recent studies examining the effect of environmental hazards on fertility claim that sperm counts are declining in certain industrialized countries. Although the validity of these findings is uncertain, most researchers agree that if there is in fact a decline in semen quality, it’s probably linked to geographic location. Now a team of California researchers has examined how exposure to specific air pollutants—ozone, nitrogen dioxide, carbon monoxide, and particulate matter smaller than 10 micrograms in diameter (PM_10_)—affects semen quality and reports a direct connection between ozone exposure and reduced sperm count **[*EHP*** **114:360–365; Sokol et al.].**

It is estimated that at least 2.1 million couples in the United States have difficulty achieving pregnancy, with male infertility responsible for 40–50% of infertility cases. Exposure to environmental toxicants that disrupt sperm production (spermatogenesis) or the function of reproductive hormones or sperm may increase the risk of male infertility.

The investigators analyzed semen samples collected from 48 men who regularly donated to a Los Angeles sperm bank between January 1996 and December 1998. Subjects were healthy, educated males between the ages of 19 and 35 who had abstained from sex for two to three days before sample collection. Also available were data on each donor’s age, date of birth, race, dates of collection, and zip code of residence at the time of first donation.

The researchers also collected air quality data gathered for ten-kilometer grid areas during the same two-year period, and assigned subjects a grid location based on their zip code at the time of first donation. Ozone, nitrogen dioxide, and carbon monoxide were measured daily, and PM_10_ was measured once every six days. Then the researchers examined the relationship between each semen sample and the air quality at 0–9, 10–14, and 70–90 days prior to its collection (human spermatogenesis is a 72-day process). They assessed semen volume, sperm concentration, and sperm motility within one hour of collection and compared it against air quality data specific to the donor.

Ozone was the only pollutant associated with changes in sperm quality. The analysis showed an inverse relationship between ozone exposure and sperm density at all points in spermatogenesis. The results remained significant after adjusting for donor age, season, and temperature.

It is known that ozone and its reaction products can cross the blood–gas barrier and enter the bloodstream, and exposure to ozone can cause oxidative stress, which has been shown to disrupt testicular and sperm function. As with smoking, ozone exposure may trigger an inflammatory reaction in the male genital tract or the formation of circulating toxic species. Both events could cause a decline in sperm concentration.

These findings support an earlier study conducted in the Czech Republic by scientists from the U.S. EPA. Young men who were exposed to elevated air pollution were more likely to have altered sperm quality than those who lived in areas with less air pollution. The authors note that the current study controlled well for potential confounders, and the connection between ozone and sperm quality is consistent across several models.

## Figures and Tables

**Figure:**
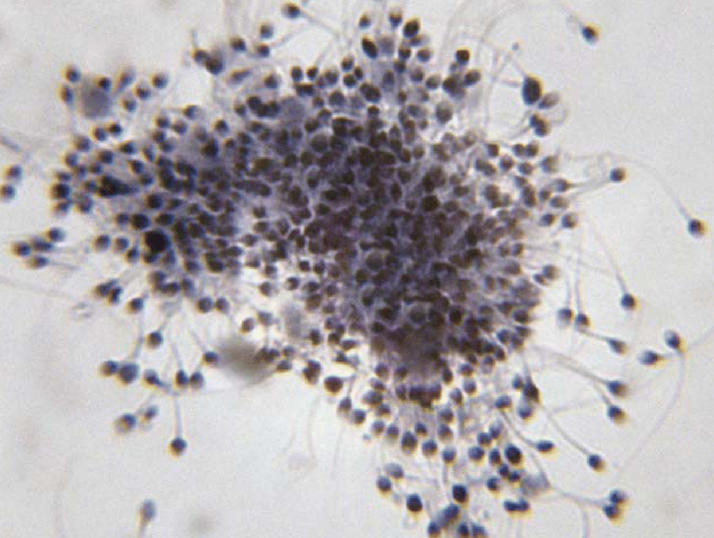
Gametes and gray skies. A new study shows significant declines in semen quality associated with exposure to ozone air pollution.

